# Association of Blood Levels of Vitamin D and Its Binding Protein with Clinical Phenotypes of Multiple Sclerosis

**DOI:** 10.3390/biomedicines11071808

**Published:** 2023-06-24

**Authors:** Suhail Al-Shammri, Arpita Chattopadhyay, Magdy Girgis Hanah, Suhail Doi, Abayomi Akanji

**Affiliations:** 1 Department of Medicine, Faculty of Medicine, Kuwait University, Kuwait City P.O. Box 24923, Kuwait; 2 Neurology Unit, Department of Medicine, Mubarak al Kabeer Hospital, Ministry of Health, Jabriya 13001, Kuwait; 3 Department of Population Medicine, Qatar University, Doha P.O. Box 2713, Qatar; 4 Department of Medical Sciences, Frank Netter School of Medicine, Quinnipiac University, Hamden, CT 06518, USA

**Keywords:** multiple sclerosis, vitamin D, 25-hydroxyvitamin D, vitamin D binding protein, Kuwait, hypovitaminosis D, relapsing remitting, drug-naïve patients

## Abstract

Background: Low vitamin D levels may synergize with changing levels of the vitamin D binding protein (DBP) to precipitate in the development and clinical progression of multiple sclerosis (MS). In this study, this hypothesis was explored in groups of Kuwaiti healthy controls and patients with different clinical phenotypes of MS. Methods: Fasting serum concentrations of 25-hydroxyvitamin D [25(OH)D] and DBP were measured in 146 healthy controls and 195 patients with MS. The latter were classified according to the duration, type, and onset of the disease and the mode of treatment. Factors such as relapse/remitting, and the use of nutritional supplements were also considered. Results: The DBP levels were significantly lower in the patients than in the controls. This was more evident in newly diagnosed drug-naïve patients than in those patients with more established MS. MS status and severity were negatively impacted by concurrently low levels of 25(OH)D and DBP. This was most clearly expressed in drug-naïve patients and in those with a disease in relapse. It was also established that the 25(OH)D level had a significant positive correlation with the duration of the disease. Conclusion: Lower levels of 25(OH)D and DBP appear to have a synergistic effect on MS status. This was most clearly demonstrated in patients who were newly diagnosed (drug-naïve) and in those patients who were in relapse.

## 1. Introduction

Multiple sclerosis (MS) is, presumably, an autoimmune-mediated demyelinating and neurodegenerative disease of the central nervous system (CNS), which usually affects young adults and causes significant irreversible neurological disability [1]. It is widely believed that both genetic and environmental factors are involved in its etiopathogenesis [2]. Further research has implicated serum vitamin D status and its binding in the circulation to the vitamin D binding protein (DBP) and genetic polymorphisms related to the DBP in the pathogenesis of MS [3]. Increasing evidence suggests that vitamin D sufficiency and related sun exposure appear to lower the risk of developing multiple sclerosis (MS) [4], and in established cases reduce relapse [5], slow progression [6], and result in fewer new lesions on the brain magnetic resonance imaging (MRI) [7].

DBP is the major plasma carrier of vitamin D and its metabolites. It is responsible for transporting much of the liver metabolite, 25-hydroxyvitamin D [25(OH)D], in the circulation system, particularly to the kidneys for further conversion to the active form 1, 25 dihydroxyvitamin D [1,25(OH)_2_D]. The latter ultimately accesses target tissues again via significant DBP transport [8]. The bioavailability of the free vitamin D metabolites is regulated by their affinity to the DBP [9]. Therefore, higher concentrations of the DBP may keep 25(OH)D and 1,25(OH)_2_D within the circulation system, while lower levels of the free vitamin D might negatively affect the potential benefits of vitamin D physiologically and, specifically, in MS patients. 

In addition to its role as a carrier protein, DBP is involved in the immune system, acting as both a chemotactic factor for neutrophils and a potent macrophage activating factor after its exposure to circulating B and T lymphocytes [8,9]. In addition, DBP functions as an actin scavenger by binding G-actin with high affinity and sequesters monomer actin released in the circulation as a result of injury and disease. Therefore, it may impact several conditions [8], possibly including MS.

The association of changing levels of DBP with the presence of MS is, however, controversial. Various studies have suggested that DBP levels are (i) low in MS patients’ cerebrospinal fluid (CSF), especially in those patients with relapsing MS [10,11]; (ii) low in the serum of patients with MS compared to the serum of healthy controls [12], and the lowest in relapsing–remitting (RRMS) patients [13,14]; (iii) higher in the CSF of patients with secondary progressive MS (SPMS) [15]; (iv) not different in the serum levels in patients with stable or active disease compared to the serum levels of controls [16,17]; and (v) according to at least one study [18], higher in RRMS patients. The implications of low DBP’s association with low circulating free vitamin D are unclear.

To address some of these controversies, we examined the associations of serum DBP with the clinical phenotypes of MS, independently and in conjunction with the serum 25(OH)D concentrations in a group of patients with MS matched with a healthy control population. The variables considered were age, gender, body mass index (BMI), duration of disease, and treatment modalities, including the use of nutritional supplements.

## 2. Materials and Methods

### 2.1. Study Design and Study Population

In this observational study, we recruited MS patients and healthy controls, approximately matching the two groups for age and sex [19], after receiving voluntary informed consent. The MS patients were diagnosed according to the McDonald criteria [20] and were followed up by our team of experienced neurologists at the national MS clinic at Mubarak Al-Kabeer Hospital in Kuwait. None of the patients had received corticosteroids during the month prior to the evaluation. The control subjects were selected from healthy individuals among the Kuwaiti population, who had no documented history of autoimmune, inflammatory, and neurologic diseases and no family history of MS. None of the controls were taking any medications. The female subjects (controls and patients) were not on estrogen supplementation.

Both groups of subjects were interviewed using a structured questionnaire to collect information about age, gender, body mass index (BMI), daily habits (including sun exposure, choice of routine outdoor dressing, routine diet, and outdoor physical activity), past medical history, and current and past medications (including dietary supplements). Moreover, the patients’ medical records were reviewed for relevant past medical history, current medications, and evidence of other chronic medical disorders requiring long-term treatment.

Thereafter, the subjects (patients and controls) had careful physical examinations. The MS patients were sub-grouped into those with established long-standing (on drug treatment) disease and those with newly diagnosed (drug-naïve) disease. They were also assigned to the clinical phenotypes of RRMS, secondary progressive MS (SPMS), and primary progressive MS (PPMS) based on the McDonald criteria [20]. The disease stage, with or without associated disability, was determined using the expanded disability status scale (EDSS) [21]. 

### 2.2. Biochemical Analysis

Fasting blood specimens were collected from all subjects and immediately centrifuged, and the serum samples were stored at −40 °C until analyzed using validated kit ELISA tests for the levels of total 25(OH)D (Immune Diagnostic Systems, Bensheim, Germany) and DBP (Quantikine, R&D systems, Minneapolis, MN, USA). The kit manufacturer’s protocols were strictly adhered to, and the assay performance characteristics (intra- and inter-assay CVs, recoveries, and analytical sensitivity and specificity) were within acceptable limits.

As per the recommendations of the clinical practice guidelines of the Endocrine Society Task Force [22], the cutoff points utilized for classification in this study were: serum 25(OH)D < 50 nmol/L—vitamin D deficiency; <25 nmol/L—severe vitamin D deficiency [23]. The other routine blood counts and clinical biochemical tests (liver and renal function) were carried out using routine Coulter Counter and Auto-analyzer (Beckman-Coulter DxC 800, Brea, CA, United States) techniques.

### 2.3. Statistical Analysis

Qualitative variables were reported as numbers and percentages, and quantitative variables as medians and interquartile ranges expressed as the 25th–75th percentile or mean and standard deviation (SD), as appropriate. The differences between the two independent groups were tested with the Mann–Whitney U test. The chi-square test and Student’s *t*-test were used for a pairwise comparison. A multivariable logistic regression model was utilized to assess associations between serum levels of 25(OH)D and DBP and MS status. The Spearman rank correlation test was used to measure the degree of association between two variables. The ODD ratios in this study were adjusted for possible confounding factors (age, gender, BMI, duration of disease, and treatment modalities, including use of nutritional supplements, mode of dressing, and daily direct sunlight exposure). Statistical analyses were carried out using Statistical Package for Social Sciences, SPSS for Windows (IBM SPSS Statistics 22, IBM Corporation, Armonk, NY, USA, 2013). A *p*-value < 0.05 was considered significant.

### 2.4. Ethical Considerations 

The study protocol and consent forms were approved by the Ethical Committee, Faculty of Medicine, Kuwait University (IORG0007925). The study was conducted in compliance with the international guidelines for human research protection, as described in the Declaration of Helsinki.

## 3. Results

The characteristics of the study population (patients and controls) are shown in Table 1.

### 3.1. Study Population 

There was a total of 195 MS patients and 146 healthy controls. The patients’ sample comprised 61.0% females and 39.0% males who were age-matched. The control population included 64.4% females and 35.6% males. The demographic and anthropometric features of both groups and the clinical phenotypes of the patients and the lifestyle habits (routine dressing, sunlight exposure) of both patients and controls are also indicated in Table 1. 

With respect to the specific treatment at recruitment into the study (Table 1), 61 (or 31%) MS patients were newly diagnosed and not taking any disease-modifying drugs (newly diagnosed (drug-naïve)), while 134 (69%) had established disease and were on disease-modifying treatment (established MS). None of the patients were on steroid treatment within the period during and a month before recruitment into the study. In addition, 81 (42%) MS patients (comprising 60 RRMS, 18 SPMS, and 3 PPMS) and 33 (25%) healthy controls reported taking regular nutritional supplements, including multivitamins and vitamin D. 

The patients were mostly young-to-middle-aged (median age 32 years), and the median age of MS diagnosis was <30 years; in addition, the median age at the onset of the symptoms was 27 years. Correspondingly, the duration of the disease at the time of recruitment was about 3 years. 

### 3.2. Clinical Features

We also established that, in the patient group at the start of the study, 126 were in remission and 65 were in relapse. The major clinical phenotypes were: 166 (85.2%) relapsing–remitting; 26 (13.3%) secondary progressive disease. Most patients (155 (79.5%)) had benign disease, with a median EDSS for all patients of just 1.5 (Table 1).

### 3.3. Serum 25(OH)D and DBP Levels

The median 25(OH)D levels (nmol/L) for both the healthy controls (28.4) and patients (27.3) were not different and fell within the vitamin D deficiency (close to severely deficient) category (Table 2). However, median serum DBP levels (µg/mL) were significantly lower in patients (163) than in controls (236; *p* < 0.001). Furthermore, the newly diagnosed (drug-naïve) MS group had lower 25(OH)D (18.9) and DBP (152) than the group with established disease (respectively 34.5, 183, *p* < 0.001). We confirmed that none of the subjects (patients and controls) had clinical or biochemical evidence of hepatic or renal disease and none of the females were on estrogen supplementation.

**Figure 1:**
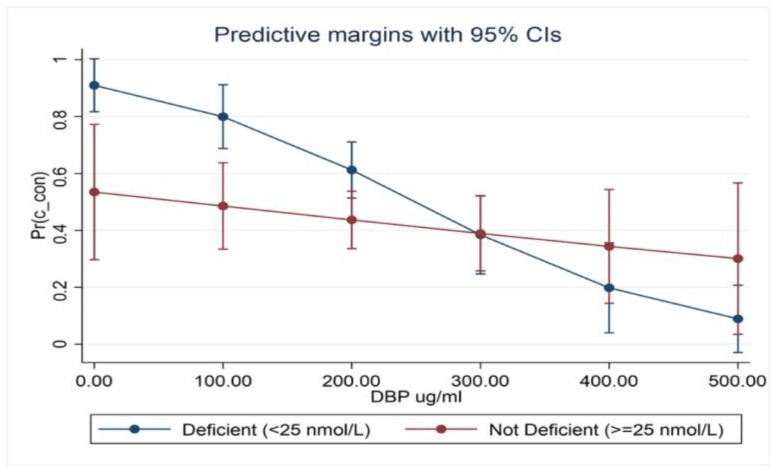
MS status predictive probability based on the logistic regression model.

We utilized logistic regression models to investigate potential interactions between serum levels of 25(OH)D and DBP and MS status in newly diagnosed (drug-naïve) patients who were not taking vitamin D supplementation. The results showed that:

- There was an interaction between 25(OH)D and DBP on MS status in severely deficient (25(OH)D < 25 nmol/L) newly diagnosed (drug-naïve) patients.
- Low serum 25(OH)D with normal DBP had no significant impact on MS status (OR 1.02; 0.54–1.92; *p* = 0.994); low serum DBP showed a better, albeit non-significant, interaction (OR 1.56; 0.87–2.82; *p* = 0.139).
- The low 25(OH)D and DBP combination demonstrated a significant association with MS (OR 2.67; 1.35–5.29; *p* = 0.005).
- Serum 25(OH)D was within strata of DBP and appeared to be more strongly associated with MS when DBP levels were reduced (OR 1.71; 0.86–3.40, *p* = 126). This relationship is best demonstrated in the marginal probabilities diagram (Figure 1).

Marginal probabilities from logistic regression in newly diagnosed (drug-naïve) patients demonstrate the interaction between vitamin D binding protein and vitamin D deficiency based on the probability of it being an MS case. The highest probability of a case was in those with both vitamin D deficiency and a lower vitamin D binding protein level.

### 3.4. Vitamin D and DBP Levels in Different MS Clinical Phenotypes

We evaluated the levels of 25(OH)D in the various clinical MS phenotypes with respect to the use or not use of vitamin D supplements in the patients’ cohort (Table 3). The results indicated as expected, that all the patients, irrespective of disease phenotype (RRMS, SPMS) and use or not of vitamin D supplementation had 25(OH)D levels in the vitamin D deficiency category Note that the patients with Primary progressive MS (PPMS) were excluded from this analysis because of their relatively small sample size (n = 3). The results showed that independent of vitamin D supplementation status, the SPMS category had significantly higher 25(OH)D levels than controls (*p* = 0.046 & 0.056 respectively), and those with RRMS (*p* = 0.025 and 0.029, respectively). This is against the background that the DBP level was similar to that seen in RRMS but marginally significantly lower than the level in the healthy controls (*p* = 0.049) in subjects taking vitamin D supplements. Contrarywise, patients with SPMS and not on vitamin D supplementation had significantly higher DBP levels than those classified as RRMS. It is noteworthy that the patients with RRMS disease had similar 25(OH)D (*p* = 0.387) but significantly lower DBP levels (*p* < 0.001) when compared to the healthy control subjects.

Table 4 indicates the influence of disease activity (remission or relapse) on 25(OH)D and DBP levels in the sub-groups of patients who were newly diagnosed (drug-naïve)—these patients are likely to be free of confounding from the use of the disease-modifying therapies. The RRMS group on remission had similar 25(OH)D but lower DBP levels than the healthy control subjects. On the other hand, the group in relapse had significantly lower 25(OH)D and DBP levels when compared to controls (both *p* < 0.05). It is noteworthy that vitamin D level was lower in relapsing than in remitting disease, although the difference did not achieve statistical significance (Table 4). 

### 3.5. Correlation of Vitamin D and DBP with Disease Severity

**Figure 2:**
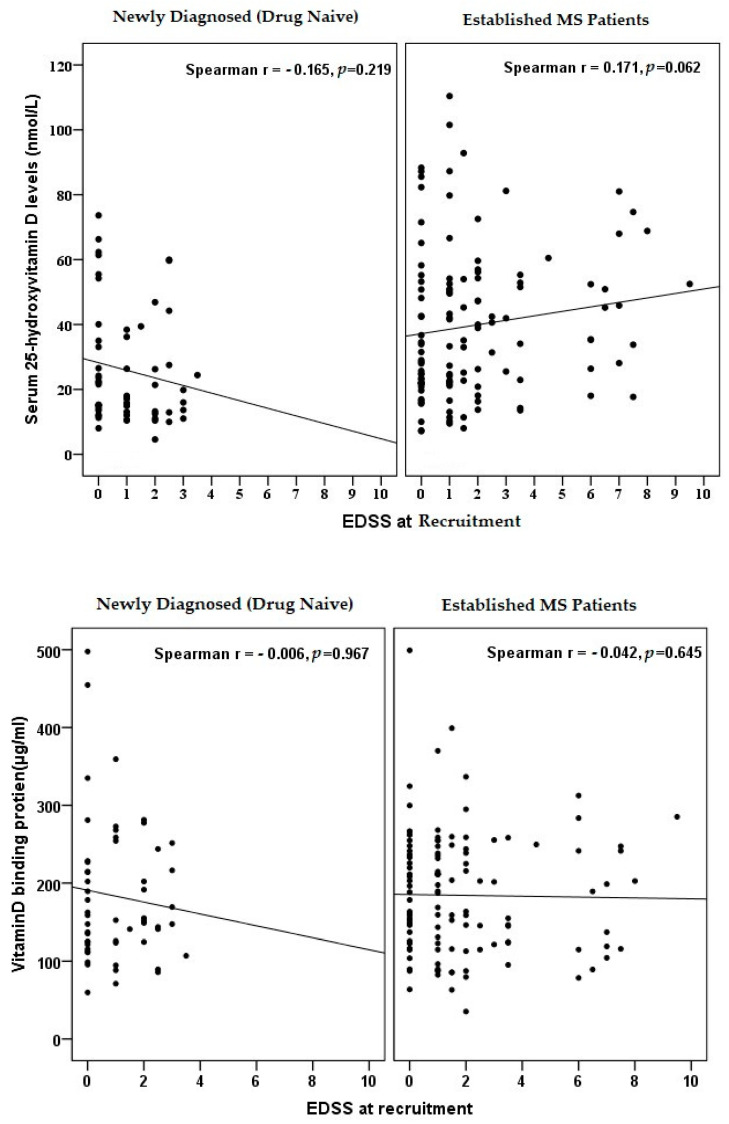
Correlation of vitamin D and Binding protein with EDSS. Serum level of 25(OH)D and VDBP are represented by black dots.

**Figure 3:**
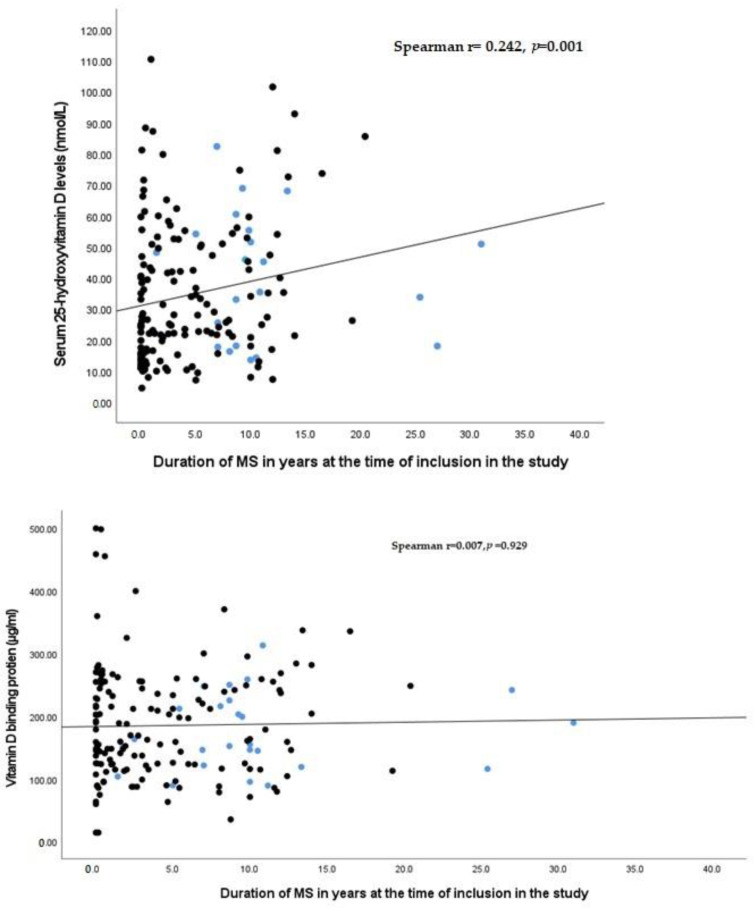
Correlation of 25(OH)D and DBP with duration of MS. Serum level of 25(OH)D and DBP are represented by dots. Black dots and blue dots represent RRMS and SPMS patients respectively.

We could not establish any significant correlation of EDSS with levels of 25(OH)D and/or DBP (Figure 2). However, serum 25(OH)D significantly positively correlated with the duration of the disease (r = 0.242; *p* = 0.001) (Figure 3). This could be ascribed to the frequent medical attention provided to patients with long-term MS with the consequence of increased awareness of the disease and the use of vitamin supplements apart from the specific medications.

## 4. Discussion

There is anecdotal evidence for increasing incidence and prevalence of MS in Kuwait. While two hospital-based studies reported an increased incidence in the last 2 decades [25,26,27], Some recent [27] and previous studies [28] suggested that Kuwait had emerged as a high-risk zone. This would be in keeping with the report by Alshubaili et al. [26], who reported that there was an apparent gradual increase in the incidence and prevalence of MS. If ultimately proven to be correct, the basis for this change might be unclear genetic, environmental, and sociocultural factors [24], perhaps ultimately acting via unexplored immunological mechanisms [25].

In this study, we have found that despite the year-round abundant sunlight in Kuwait, there is widespread vitamin D deficiency, as seen in both groups of healthy control subjects and patients with MS. The study also suggested an association of low 25(OH)D and low DBP with the development of MS, most clearly seen in the cohort of patients who were newly diagnosed (drug-naïve). 

Other important findings from the study are: (i) the serum DBP level was inversely associated with MS prevalence; (ii) there is an interaction between circulating DBP and 25(OH)D and clinical MS; (iii). high DBP levels appear to attenuate the association of clinical MS status with concurrent vitamin D deficiency; (iv). there appears to be a > 2.5 X increase in the likelihood of MS development when serum levels of both DBP and 25(OH)D are low, and (v). the association of vitamin D with the development of MS appears strongest when DBP levels are low.

To the best of our knowledge, the synergism we have described between 25(OH)D and its carrier protein (DBP) in the development of MS and its clinical phenotypes is novel. at least in our relatively homogeneous Arab population. The immediate deduction is that higher DBP protects against hypovitaminosis D-mediated MS risk. Whether this is a metabolic interaction or is precipitated by the other well-described independent effects of DBP is currently uncertain but deserves further study because of its potential therapeutic implications.

The plausibility of interaction between DBP and its ligand 25(OH)D to influence exposure risk is however not new. It has been observed previously in other biological systems including cancer [29].

At the molecular level, the uptake of the vitamin D-DBP complex in various organs and tissues [30]) is mediated by the plasma membrane receptor megalin. This is particularly important in the renal proximal tubules for resorption of DBP-25(OH)D from the glomerular filtrate and subsequently conversion of 25(OH)D to the bioactive form, 1,25(OH)_2_D. It has been demonstrated that megalin knockout mice are incapable of resorbing 25(OH)D–DBP complex from urine resulting in loss of 25(OH)D and profoundly low circulating 25(OH)D and 1,25(OH)_2_D levels. It is therefore possible that higher circulating DBP promotes greater renal tubule DBP resorption and higher bioavailable 1,25(OH)_2_D to target tissue and mitigate a putative MS risk related to low circulating 25(OH)D levels. The presence of megalin in the brain will provide some evidence for the role of DBP in increasing the neural bioavailability of vitamin D and subsequent protection from demyelination. 

In the current study, the plasma level of DBP was significantly lower in patients than in the healthy controls and lowest in the RRMS-relapse group. This is consistent with previous observations [12,13,14]. Some other studies reported no change in circulating DBP in relapsing MS during remission and relapse [16] and during remission [12] as compared to healthy controls, while at least one study [18] reported higher DBP levels in RRMS. We believe that these discrepant observations may be due to, among other variables, issues with sample population size and heterogenicity (our population was demographically and socioculturally relatively homogenous), and DBP analytical methods [31].

Lower DBP and 25(OH)D levels, as observed in our study, might indicate that free and bioavailable vitamin D levels are also low, although we did not specifically calculate a free vitamin D index [13]. Both those latter studies indeed did demonstrate that low levels of DBP influence MS risk, even at the pre-symptomatic phase. It seems reasonable to suggest that the combination of low DBP and low 25(OH)D will attempt to preserve the free vitamin D index for physiologic ubiquitous vitamin D function. However, with intensified tissue inflammation and/or lack of disease modification with drugs [as seen in the newly diagnosed (drug-naïve) patients or those with MS relapse], continuing reductions in 25(OH)D and DBP (possibly as part of the acute phase response), will upset the vitamin D/DBP equilibrium, with changes in the free vitamin D index and tendency towards the clinical expression of MS. This is however speculative and subject to confirmation. What is non-controversial is that DBP potentially influences the immune system [32] and may have a favorable impact on immune regulation independent of, and in association with the immunoregulatory functions of vitamin D [measured as 25(OH)D] in the pathogenesis of MS. Certainly, the genetic polymorphism of DBP itself (not explored on this study) may also play a role in this interaction.

There have been a few reports on the potential role of brain DBP (as measured by CSF analyses) in the pathogenesis of MS. Some CSF proteomic studies found lower levels of DBP in MS [10,11] in relapse and in comparison, to controls with other neurologic disorders. Other recent studies reported lower DBP CSF levels during the acute phase of demyelinating attacks in MS patients [33], which appears to mirror observations in the peripheral circulation [12,14]. However, these observations are not consistent [16]. 

This study could not support reports from some other studies [17] of significant associations between vitamin D levels and measures of disability (EDSS). We believe that such associations might be possible with larger subject numbers and will form the basis of our continuing studies. DBP levels are significantly influenced by genetic polymorphism and being acute phase proteins might also be influenced by medications and concomitant physical illnesses. All these, with vitamin D nutriture (that can be influenced by diet in the chronically ill, even in a relatively high-income population) will affect the links between vitamin D, DBP, and MS prevalence and phenotypes. Our study did not specifically evaluate the interplay of all these issues and should possibly be considered a pilot. There are ongoing efforts to investigate in more detail these mechanistic issues. Nonetheless, this study had several strengths, including a larger sample size, the inclusion of both drug-naïve patients and those who are on disease-modifying therapies, and analyzing a patient population that included different MS phenotypes and individuals in remission and/or relapse. One important limitation however is the relatively small number of SPMS and the lack of measurement of free vitamin D and indicators of DBP genetic polymorphism.

## 5. Conclusions

The study has demonstrated that 25(OH)D deficiency is common in both Kuwaiti patients with MS and apparently healthy controls, probably because of a wide variety of social, environmental, and genetic factors. We have suggested an association between low levels of 25(OH)D and DBP and the presence of MS, which appeared strongest in the drug-naïve MS patients, whose biochemical measurements could not have been confounded by medications. Additionally, MS patients in relapse had relatively low 25(OH)D and DBP levels. We could not establish associations between serum 25(OH)D and DBP concentrations and disability in MS. We suggest that the homeostatic regulation of the relationship between circulating 25(OH)D level and its binding protein, DBP, may be impaired in the pathogenetic process involved in the onset of MS and its progression into distinct clinical phenotypes. This may have been triggered by yet unknown genetic and environmental factors and which will form the basis for our further studies.

## Figures and Tables

**Table 1:** The characteristics of the study population (patients and controls) ([24]).

	Controls (n)	Patients with MS (n)			*p* *	*p* ^$^	*p* #
		All	Established Patients	Newly Diagnosed (Drug-Naïve) Patients			
Number (n)	146	195	134	61			
Age (in years) mean (SD)	33.8 (9.5)	33.2 (10.4)	34.4 (11.0)	30.8 (8.3)	0.589 ^b^	0.665 ^b^	0.029 ^b^
Gender					0.526 ^a^	0.672 ^a^	0.466 ^a^
Male	52 (35.6)	76 (39.0)	51 (38.1)	25 (41.0)			
Female	94 (64.4)	119 (61.0)	83 (61.9)	36 (59.0)			
BMI (kg/m^2^)	28.7 (10)	28.6 (12.5)	28.8 (11.5)	28.2 (10.5)	0.359 ^c^	0.241 ^c^	0.933 ^c^
25–29.9 (overweight)	50 (36.8)	59 (31.9)	39 (30.5)	20 (35.1)			
>30 (Obese)	49 (36.0)	62 (33.5)	42 (32.8)	20 (35.1)			
Daily direct sunlight exposure (minutes)					<0.001 ^a^	<0.001 ^a^	0.008 ^a^
<10	36 (26.1)	62 (31.8)	37 (27.6)	25 (41.0)			
10–15	32 (23.2)	93 (47.7)	72 (53.7)	21 (34.4)			
15–30	35 (25.4)	25 (12.8)	17 (12.7)	8 (13.1)			
>30	35 (23.4)	15 (7.7)	8 (6.0)	7 (11.5)			
Mode of dressing					0.654 ^a^	0.980 ^a^	0.026 ^a^
Traditional/Western (Exposed Face/Arms	112 (83.0)	158 (81.0)	116 (86.6)	42 (68.9)			
Fully covered eyes/hands Exposed	23 (17.0)	37 (19.0)	18 (13.4)	19 (31.1)			
MS Subtype							
Relapsing–remitting (n, %)	-	166 (85.2)	105 (78.4)	61 (100)			
Secondary progressive (n, %)	-	26 (13.3)	26 (19.4)	-			
Primary progressive (n, %)	-	3 (1.5)	3 (2.2)	-			
Age at recruitment year (median *)	-	32 (27–38)	33 (27–41)	31 (26–36)			
Age at diagnosis, year (median *)	-	28 (21–34)	27 (20–33)	31 (26–36)			
Age at onset, year, (median *)	-	27 (20–32)	26 (19–32)	29 (22–33)			
Duration of disease from onset of symptoms, year (median *)	-	3 (1–8)	5 (2–10)	2 (1–7)			
EDSS score (median *)	-	1.5 (0–2.5)	1.5 (0–3)	1 (0–2)			
Annualised relapse rate (median *)		3 (1–5)	4 (2–7)	0.1 (0.1–0.2)			

n: number of subjects. Numeric data are expressed as the mean (SD), medians * (25th–75th percentile), and percentage (%), as appropriate; *p*-values are generated via ^a^ chi-square test, ^b^ Student’s *t*-test, ^c^ Mann–Whitney U test for pairwise comparisons, as appropriate; *p* * Controls vs. All MS patients; *p* ^$^ Controls vs. Established patients; *p* # Controls vs. Newly diagnosed (drug-naïve) patients.

**Table 2:** Levels of 25(OH)D and DBP in the study population.

	Controls (n)	Patients with MS (n)			*p* ^a^	*p* ^b^	*p* ^c^	*p* ^d^
		All	Established MS	Newly Diagnosed (Drug-Naïve)				
n	146	195	134	61				
25(OH)D (nmol/L)	28.4 (18.0–50.4)	27.3 (17.0–50.8)	34.5 (22.1–52.7)	18.9 (13.0–35.9)	0.82	0.081	0.001	<0.001
DBP (µg/mL)	236 (152–288)	163 (123–241)	183 (123–241)	152 (117–235)	<0.001	<0.001	<0.001	0.401

n: the number of subjects. Numeric data are expressed as the medians (25th–75th percentile); *p*-values are generated via the Mann–Whitney U test for a pairwise comparisons. *p* ^a^ All MS patients vs. Controls; *p* ^b^ Established MS vs. Controls; *p* ^c^ New Diagnosed (drug-naïve) vs. Controls; *p* ^d^ Newly Diagnosed (drug-naïve) vs. Established MS patients.

**Table 3:** Serum levels of 25(OH)D and DBP in different MS phenotypes vs controls.

	Healthy Controls	Relapsing-Remitting	*p* ^a^	Secondary Progressive	*p* ^b^	*p* ^c^
All Subjects (n)	146	166		26		
Subjects on Vitamin D supplement	33	60		16		
25(OH)D (nmol/L)	28.4 (18.0–50.4)	25.7 (16.0–47.8)	0.387	45.5 (22.0–59.2)	0.046	0.025
DBP (µg/mL)	236 (152–288)	162 (123–239)	<0.001	194 (127–246)	0.049	0.508
Subjects not on Vitamin D supplement	n = 112	n = 101		n = 10		
25(OH)D (nmol/L)	26.2 (16.7–35.0)	22.1 (13.0–36.3)	0.051	35.4 (19.4–64.7)	0.056	0.029
DBP (µg/mL)	246 (161–299)	159 (120–248)	<0.001	247 (222–271)	0.801	0.011

Values are expressed by Median (25th–75th percentile). *p*-values are generated using Mann–Whitney U test for pairwise comparisons ^a^ Relapsing-Remitting vs. Controls; ^b^ Secondary Progressive vs. Controls; ^c^ Relapsing-Remitting vs. Secondary Progressive. Note that Numbers may not add up to the total due to missing data.

**Table 4:** 25(OH)D and DBP levels in controls and newly diagnosed (drug-naïve) patients in relapse and remission.

	Controls	MS Relapse	*p* ^a^	MS Remission	*p* ^b^	*p* ^c^
N	146	36		25		
25(OH)D (nmol/L)	25.4 (16.3–33.5)	16.0 (12.5–26.5)	0.028	22.5 (14.1–47.2)	0.198	0.058
DBP (µg/mL)	247 (165–303)	155 (113–244)	<0.001	151 (118–233)	0.001	0.318

Values are expressed by the median (25th–75th percentile). *p*-values are generated using the Mann–Whitney U test for pairwise comparisons. *p* ^a^ patients having relapse vs. controls, *p* ^b^ patients having remission vs. controls; *p* ^c^ patients having remission vs. relapse.

## Data Availability

The database generated and/or analyzed during the current study are not publicly available due to ethical considerations but are available from the corresponding author on reasonable request.
